# Maternal and perinatal factors associated with hospitalised infectious mononucleosis in children, adolescents and young adults: record linkage study

**DOI:** 10.1186/1471-2334-11-51

**Published:** 2011-02-28

**Authors:** Imran Mahmud, Omar A Abdel-Mannan, Clare J Wotton, Michael J Goldacre

**Affiliations:** 1 Clinical Medicine, Somerville College, University of Oxford, Oxford, OX2 6HD, UK; 2 Clinical Medicine, St John's College, University of Oxford, Oxford, OX1 3JP, UK; 3 Unit of Health-Care Epidemiology, Department of Public Health, University of Oxford, Old Road Campus, Old Road, Oxford OX3 7LF, UK

## Abstract

**Background:**

There is current interest in the role of perinatal factors in the aetiology of diseases that occur later in life. Infectious mononucleosis (IM) can follow late primary infection with Epstein-Barr virus (EBV), and has been shown to increase the risk of multiple sclerosis and Hodgkin's disease. Little is known about maternal or perinatal factors associated with IM or its sequelae.

**Methods:**

We investigated perinatal risk factors for hospitalised IM using a prospective record-linkage study in a population in the south of England. The dataset used, the Oxford record linkage study (ORLS), includes abstracts of birth registrations, maternities and in-patient hospital records, including day case care, for all subjects in a defined geographical area. From these sources, we identified cases of hospitalised IM up to the age of 30 years in people for whom the ORLS had a maternity record; and we compared perinatal factors in their pregnancy with those in the pregnancy of children who had no hospital record of IM.

**Results:**

Our data showed a significant association between hospitalised IM and lower social class (p = 0.02), a higher risk of hospitalised IM in children of married rather than single mothers (p < 0.001), and, of marginal statistical significance, an association with singleton birth (p = 0.06). The ratio of observed to expected cases of hospitalised IM in each season was 0.95 in winter, 1.02 in spring, 1.02 in summer and 1.00 in autumn. The chi-square test for seasonality, with a value of 0.8, was not significant.

Other factors studied, including low birth weight, short gestational age, maternal smoking, late age at motherhood, did not increase the risk of subsequent hospitalised IM.

**Conclusions:**

Because of the increasing tendency of women to postpone childbearing, it is useful to know that older age at motherhood is not associated with an increased risk of hospitalised IM in their children. We have no explanation for the finding that children of married women had a higher risk of IM than those of single mothers. Though highly significant, it may nonetheless be a chance finding. We found no evidence that such perinatal factors as birth weight and gestational age, or season of birth, were associated with the risk of hospitalised IM.

## Background

Infectious mononucleosis (IM) can follow a pathologically strong immune response to primary infection with Epstein-Barr Virus (EBV), mainly during adolescence or young adulthood[1]. The majority of individuals have a primary infection with EBV during infancy and childhood, and are asymptomatic or only experience a mild clinical course[2,3]. Some individuals who are not infected in childhood are subsequently infected in adolescence or adulthood, leading to more severe disease. For example, a recent study of university students in Edinburgh found that three-quarters were EBV seropositive at entry to university; that, of the quarter who were seronegative, almost half experienced EBV sero-conversion over the following three years; and that, of these, 25% developed IM[4]. Two-thirds of the IM cases, but only one tenth of the asymptomatic primary EBV infections, were statistically attributable to sexual intercourse[4]. At least in student populations, sexual intercourse and intimate kissing are important factors in the transmission of EBV infections that lead to IM.

Epidemiological and laboratory data show that EBV infection is also associated with several other diseases. EBV was first identified in Burkitt's lymphoma cells[5]. EBV infection has been known for many years to be associated with nasopharyngeal carcinoma in some parts of the world, [6] and with Hodgkin's disease (HD),[7-9] associations that have been amply confirmed[3]. More recently, it has been demonstrated that IM is a risk factor for multiple sclerosis (MS)[10-13]. Factors that are relevant to the epidemiology of IM may also have some relevance to the epidemiology of Hodgkin's disease and MS, and vice versa.

There has been considerable interest in recent years in the influence of maternal and perinatal factors on the subsequent development of disease in later life[14]. Much of the interest has focused on subsequent chronic non-infectious diseases, such as hypertension, coronary heart disease and diabetes,[15-17] rather than acute infectious disease. Specifically, there is little or no information on whether perinatal factors might have any influence on the development of IM.

There are reasons to consider the possibility that perinatal and/or other early life factors might influence the risk of IM. First, there is the fact that many individuals are infected with EBV very early in life, while others are not and have an increased risk of IM later. Second, Purtilo and Sakamoto reported that reactivation of EBV commonly occurs in normal pregnant women and commented that "the impact of pregnancy on outcomes of EBV infections has not been thoroughly evaluated" in respect of either the mother or child[18]. There is still a paucity of research in this area. Third, migration patterns for MS, between high and low risk countries, show that the risk of MS is substantially determined by place of residence in early life rather than later[19,20]. Fourth, there are reasons to think that pregnancy-related or other early life factors may influence the development of MS in some people: in particular, there is increasingly strong evidence that the distribution of season of birth in people with MS differs from that in the general population[21,22]. There is an excess of spring births, albeit a numerically modest excess, among people with MS with the implication that pregnancy-associated factors may be relevant to the risk of MS. There is also some evidence of season of birth effects in HD with a slight excess of spring births in young people with HD[23].

For these reasons, we decided to use the Oxford record linkage study (ORLS) to study perinatal factors in people who developed IM, as part of a wider programme of work studying the influence of perinatal factors on the subsequent development of disease in the offspring[24-26]. The ORLS dataset has already been used, in previous studies, to demonstrate that there is an increased risk of MS and of HD in individuals following admission to hospital with IM in the Oxford area[27,28].

## Methods

The Oxford record linkage study (ORLS) includes abstracts of birth registrations, maternities and in-patient hospital admission records, including day case care (ie admission to hospital for care without overnight stay), for all subjects in a defined geographical area of South East England. The maternity data covered all National Health Service (NHS) hospitals in two health districts from 1970 to 1989 (in 1989 detailed data collection on maternity in the ORLS stopped after reforms by the government to increase the uniformity of NHS data collection systems). Cases of hospitalised IM were identified using in-patient and day case admission data in the ORLS for all clinical specialties and from all districts covered by the ORLS including those that did not collect maternity data. These data covered the two health districts from 1970 to 1999 (population 0.9 million in 1999); a further four adjacent districts from 1970-1991 (total population 1.9 million); and all eight districts of the former Oxford region from 1991-1999. The maternity data were extracted from maternity records by clerical staff, trained at the ORLS by senior medical staff. In the 30-year period covered by this study, the abstracts relating to the same individual were linked as part of the Oxford region's NHS health information system. Similarly, the records of each mother and her offspring were routinely linked. From these sources, we identified cases of hospitalised IM up to the age of 30 years in people for whom the ORLS had a maternity record. IM in the mothers was identified by record linkage of each mother's maternity record to hospital admissions for the mother before and after the pregnancy from 1963 to 1999.

Exclusions from the analysis of the maternity dataset included 985 abortions, 1560 stillbirths and 1567 neonatal deaths within 30 days of birth. In 289 maternities, the birth weight was recorded as less than 1000 g - these were also excluded because most of these records had implausibly low values and/or missing data for many of the risk factors investigated. None of these 289 excluded babies had a subsequent record of IM. After exclusions, records of 248 659 children remained. Some data items such as social class, mother's smoking and breast feeding at the time of discharge from hospital were not collected until 1975.

We accepted, as a case of IM, each person in the ORLS who had a hospital discharge record that included the International Classification of Diseases (ICD) code for IM. The codes used were 075 in the eighth and ninth revision of the ICD and B27 in the tenth. The occupation of the head of the mother's household was recorded based on husband's occupation, or the mother's occupation if single and working, or the mother's father's occupation if not. It was recorded contemporaneously on the mothers' hospital records, obtained by trained interviewers at hospital admission; and was subsequently coded by trained coders as occupational social class in the five standard groups then used in English national statistics (social class one is the most advantaged socio-economic group, and social class five the most deprived).

The duration of follow-up for the offspring ranged from 30 years for those born in 1970 to 10 years for those born in 1989, with a mean follow-up duration of 18 years. We analysed all cases of IM together and then split the analysis into those aged 10 and under and those aged 11 years and over. We did this, first, because we have 10 years of follow-up of all infants, but a variable length of follow-up thereafter; and, second, because we were particularly interested in cases of late onset IM.

Statistical methods used include chi-squared tests to assess the significance of associations between each individual perinatal risk factor and IM in the offspring, and logistic regression modelling to investigate risk factors that had significant independent associations with IM. Statistical significance was measured at the standard 5% level. When using logistic regression, all variables that were significant (P < 0.05) in the univariate analysis were included in the initial model and the variables that were not significant were removed before running the initial model. Thereafter, each of the variables that were not significant in the univariate analysis was re-introduced into the model, one at a time. The purpose of this was to test whether any variable, if not significant in univariate analysis, became significant when modelled with other significant variables. Missing data were excluded only for those terms that were included in the logistic regression model.

In order to test the hypothesis that the season-of-birth distribution for people with IM may differ from that in the general population, we also used a much larger ORLS dataset. This dataset covers all records in the ORLS from 1963-1999, not just those linked to maternity records in the smaller area from 1970-1989. We analysed season of birth in patients who were born in the UK (to avoid confounding with the place of birth of people born overseas, e.g. on the Indian subcontinent). We calculated the 'expected' number of births of IM patients in each month by applying the monthly distribution of all births in the general UK-born population in the ORLS to the number of people with IM. We did this with adjustment for year of birth, sex, and for differences in the number of days in different months. We compared the expected number of births in each month with the observed number, and expressed the result as a ratio of monthly observed to expected. We used a chi square test for heterogeneity to test for differences between individual months and between four seasons of winter (December, January, February), spring (March, April, May), summer (June, July, August) and autumn (September, October, November).

To provide contextual information on the incidence of hospitalised IM in the region covered by the study, we analysed trends over time in population-based admission rates using the whole ORLS dataset.

The English NHS Central Office for Research Ethics Committees approved the current work programme of analysis using the linked dataset (reference number 04/Q2006/176).

## Results

There were 225 people with a maternity record in the ORLS and with a subsequent admission for IM. 69 of the 225 people with hospitalized IM (31%) were aged 10 years or less at the time of admission for IM (39 males, 30 females); and 156 (69%) were aged 11 and over (74 males, 82 females). We noted that, although there were more male than female cases in the age group under ten, and more females than males in the age group aged 10-19, these findings did not reach statistical significance. Numbers of people admitted for IM were highest in the 15-19 age group, and a larger number of over 20s were admitted than children under 5 (Table 1). This age profile of patients contrasts with that of EBV infection, generally, which frequently occurs in infancy.

**Table 1:** Number of people admitted to hospital for infectious mononucleosis (IM), based on age at admission and sex.

Age at IM admission	Male (%)	Female (%)	Total (%)
0-4 years	9 (8.0)	6 (5.4)	15 (6.7)
5-9 years	25 (22.1)	18 (16.1)	43 (19.1)
10-14 years	15 (13.3)	21 (18.7)	36 (16.0)
15-19 years	47 (41.6)	53 (47.3)	100 (44.4)
20+ years	17 (15.0)	14 (12.5)	31 (13.8)
			
Total	113 (100)	112 (100)	225 (100)

Chi square, comparing males and females in each age group, = 3.39, 4 df, p = 0.50).

There was no significant association between IM in the child and maternal IM, smoking, parity, ABO blood group and rhesus status (Table 2). In most analyses, we grouped parity as either first-born or subsequent-born. However, we show parity in greater detail in Table 3 to demonstrate that there were no important differences, in detail, between those with and without IM. Overall, IM was more common in children of younger than of older mothers; the same was observed in those with IM aged 11 and over, though differences were marginally significant (p = 0.04) and fairly small (Table 2). There was an increased risk of IM amongst lower social classes though, again, the differences were fairly small (Table 2). IM was significantly more common in children of mothers who were married than in children of mothers who were single (p < 0.001): ninety seven per cent of children admitted with IM had a married mother compared with 90% of children who were not admitted with IM (Table 2).

**Table 2:** Associations between mothers' characteristics and IM in the child

		Number and % of each group of children						Chi sq (df) with p value below)	
		IM all ages		IM 11+		No IM		All IM (vs no IM)	IM aged 11+ (vs no IM)
		No.	%	No.	%	No.	%		
									
**Maternal IM**	no	222	98.7	154	98.7	243831	98.2	0.3(1)	0.3(1)
	yes	3	1.3	2	1.3	4554	1.8	0.6	0.6

**Maternal age**	14-24	88	39.1	66	42.3	86456	34.8	2.4(1†)	4.4(1†)
	25-34	124	55.1	82	52.6	142815	57.6	0.1	0.04
	35+	13	5.8	8	5.1	18839	7.6		

**Maternal social class**	1,2 (higher)	53	28.3	40	28.8	68191	35.8	5.6*(1†)	3.5(1†)
	3	90	48.1	67	48.2	86779	45.6	0.02	0.06
	4,5 (lower)	44	23.5	32	23	35466	18.6		

**Maternal marital status**	not married	6	2.7	4	2.6	23933	9.7	12.6*(1)	9.0*(1)
	married	219	97.3	152	97.4	224042	90.4	0.0004	0.003

**Maternal smoking**	no	72	72	47	71.2	110889	76.4	1.1(1)	1(1)
	yes	28	28	19	28.8	34217	23.6	0.3	0.3

**Parity**	0	87	38.7	61	39.1	104123	42	1(1)	0.5(1)
	1 or more	138	61.3	95	60.9	144076	58.1	0.3	0.5

**Maternal blood group**	A	93	50.3	64	47.8	100917	48.9	0.1(1)	0.07(1)
	O	92	49.7	70	52.2	105299	51.1	0.7	0.8

**Maternal rhesus status**	positive	171	82.2	123	82.6	196481	83.2	0.1(1)	0(1)
	negative	37	17.8	26	17.5	39768	16.8	0.7	0.8

* Significant difference, with exact p value given

† Chi square for trend

df = degrees of freedom

**Table 3:** Distribution of mothers' parity at the time of birth of people without and with eventual IM

Parity	Without IM		With IM	
	No.	%	No.	%
0	104210	42.0	87	38.7
1	88997	35.9	84	37.3
2	36081	14.5	37	16.4
3	12101	4.9	9	4.0
4 or more	6897	2.8	8	3.6
				
Total	248199	100.0	225	100.0

Children with IM were more likely to have been singletons than others, but this finding did not quite reach statistical significance (P = 0.2 overall, 0.06 among those aged 11 years and over, Table 4). We found no significant association between IM and birth weight, gestational age, breastfeeding, caesarian birth, presentation at delivery or Apgar scores at 1 and 5 minutes after delivery. Children with IM were significantly more likely to have had a forceps delivery than a child without IM, both in the all-ages analysis (p = 0.008) and in that for children with IM aged 11 years and over (p = 0.02). There was a borderline significant association between pre-eclampsia and IM (p = 0.07) (Table 3).

**Table 4:** Associations between characteristics of the births and IM in the child

	Number and % of each group of children							Chi sq (df) with p value below	
									
		IM all ages		IM 11+		No IM		All IM (vs no IM)	IM aged 11+ (vs no IM)
		No.	%	No.	%	No.	%		
									
**No. of babies delivered**	1	223	99.1	156	100	243046	97.8	1.7(1)	3.5(1)
	2+	2	0.9	0	0	5388	2.2	0.2	0.06
									
**Birth weight (g)**	1000-2999	61	27.2	44	28.4	58492	23.6	1.7(2)	2.5(2)
	3000-3999	144	64.3	96	61.9	168005	67.9	0.4	0.3
	4000-5499	19	8.5	15	9.7	21132	8.5		
									
**Gestational age**	24-37w	19	9	11	7.4	21893	10.1	0.7(2)	2.2(2)
	38-41w	170	80.2	119	80.4	173698	80.4	0.7	0.3
	42-47w	23	10.9	18	12.2	20544	9.5		
									
**Breastfeeding**	no	31	25.6	21	27.6	50935	30.3	1.2(1)	0.3(1)
	yes	90	74.4	55	72.4	117274	69.7	0.3	0.6
									
**Caesarian birth**	no	209	92.9	147	94.2	223584	92.6	0(1)	0.6(1)
	yes	16	7.1	9	5.8	18009	7.5	0.8	0.4
									
**Forceps**	no	182	80.9	126	80.8	209918	86.9	7.1*(1)	5.1*(1)
	yes	43	19.1	30	19.2	31675	13.1	0.008	0.02
									
**Presentation**	vertex	112	93.3	72	96	158190	95	0.7(1)	0.2(1)
	other	8	6.7	3	4	8303	5	0.4	0.7
									
**Apgar 1 score**	1 to 5	19	9.6	15	10.6	21337	9.5	2.1(2)	2(2)
	6 to 8	65	33	47	33.1	64404	28.5	0.4	0.4
	9 to 10	113	57.4	80	56.3	140154	62		
									
**Apgar 5 score**	1 to 5	0	0	0	0	884	0.6	0.6(2)	0.4(2)
	6 to 8	3	3	2	2.9	4226	2.8	0.7	0.8
	9 to 10	97	97	66	97.1	148738	96.7		
									
**Gender**	male	113	50.2	74	47.4	127716	51.4	0.1(1)	1(1)
	female	112	49.8	82	52.6	120711	48.6	0.7	0.3
									
**Pre-eclampsia**	no	195	86.7	133	85.3	224165	90.3	3.3(1)	4.4*(1)
	yes	30	13.3	23	14.7	24220	9.8	0.07	0.04

* Significant difference, with exact p value given

df = degrees of freedom

An association with marital status persisted after multivariate adjustment: IM was less common in children of single mothers than in children of married mothers (odds ratio, single to married, 0.36, 95% confidence interval 0.16-0.80) after adjustment for maternal age, parity and social class. The association between marital status and IM seems to be independent of either parity or social class, and is illustrated in Table 5. As Table 5 shows, the percentages of children with IM are systematically higher for those whose mothers were married, regardless of parity (summarised as first-born or subsequent child) and regardless of social class (summarised as 1 and 2, the most favoured social class, to 4 and 5, the most deprived). However, numbers of cases of IM in the unmarried category are very small, and, though the differences were systematic they were not generally statistically significant within subgroups.

**Table 5:** Percentage in each group with IM, by marital status, and numbers on which percentages are based (n/N)

Group	Percentage with IM		number with IM/N in subgroup	
	
	Married	Not married	Married	Not married
First born	0.09	0.02	84/88683^1^	3/15324^1^
Younger siblings	0.10	0.04	135/135393^2^	3/8566^2^
				
Social class* 1,2	0.08	0.00	53/66358^3^	0/1821^3^
Social class* 3	0.11	0.02	89/82495^4^	1/4286^4^
Social class* 4,5	0.13	0.04	43/32917^5^	1/2558^5^

* Data only available for part of the study period

Chi square values (with Yates' correction), comparing hospitalised IM in offspring of mothers who were married or not married, ^1^7.95, p = .005; ^2^2.88, p = .09; ^3^0.61, p = 0.44; ^4^2.05, p = 0.15; ^5^0.95, p = 0.33.

Multivariate analysis showed that pre-eclampsia and use of forceps during delivery were not independently associated with an increased risk of IM, after controlling for year of birth and social class.

In the analyses of season of birth, there was no significant association in the dataset of the 225 patients on whom we had a maternity record. In the full ORLS dataset (see Method), there were 1695 people with a record of hospitalised IM. The ratio of observed to expected cases of IM in each season was 0.95 in winter, 1.02 in spring, 1.02 in summer and 1.00 in autumn. The chi-square test for seasonality, with a value of 0.8, was not significant.

The analyses of trends show that there were no major changes over time - for example, admission rates in the ORLS area were 4.4 per 100,000 population in 1975, 4.4 in 1985 and 4.7 in 1995.

## Discussion

### Strengths and limitations

Strengths of this study are that data collection was prospective, undertaken in a large and well-defined population, over a period of 30 years, including around 250,000 births, and recall biases are impossible. Data concerning perinatal risk factors, and subsequent IM, were collected independently. They were brought together by record-linkage, and therefore data about risk factors could not have been influenced by knowledge of the study outcome (IM) or by the kinds of interviewer, recall or attribution bias that can handicap case-control studies based on interviewing patients.

Despite the large study population, the total number of cases of IM identified was a modest 225. This limits the power of the study. To our knowledge, there are no other reports in the published literature concerning perinatal factors and subsequent IM. Cases of IM not requiring hospitalisation will have been missed by this study. IM is diagnosed primarily based upon a clinical picture of symptoms, peripheral blood smear, and heterophile (Monospot) antibody test. It seems likely that hospitalised cases are more likely than those that do not warrant admission to have had confirmatory tests done to establish the diagnosis with certainty. However, we do not have data on the diagnostic criteria used in, or the clinical features of, the study population. We had to accept a coded diagnosis on the hospital discharge abstract. Current privacy regulations preclude checking the actual medical records of the patients for further detail.

There are some gaps in the data collection: smoking behaviour and social class were not routinely collected for a few years of the study. We could not identify records of children who were diagnosed with IM after moving away from the ORLS region, lowering our observed incidence of IM. It is certain that our observed IM incidence is lower than the true incidence of IM. However, the influence of perinatal risk factors, when comparing children with and without IM, should not be biased unless migration itself is associated with both the risk of subsequent IM and putative perinatal risk factors. We found very few significant associations. It is theoretically possible, though we think unlikely, that associations have been missed as a result of unmeasured confounding, i.e. that a true association has been masked by confounding factors that act in equal and opposite directions to a true cause-and-effect association.

Although cases of IM requiring hospital admission are infrequent, they are likely to represent people at the severe end of the clinical spectrum. If perinatal and maternal factors affect the risk of IM, they are more likely to affect those with severe disease. Those with symptoms severe enough to warrant hospital admission may also have the strongest reactions to primary EBV infection, which in turn, may represent individuals who are more susceptible to diseases where EBV is thought to play an aetiological role, notably HD and MS[29]. We hope that others will be stimulated to publish results from similar databases and, if future individual studies are limited in size, we hope that our data and others could be pooled to produce meta-analyses.

### Delayed EBV infection, IM, HD and MS

In the majority of individuals, primary EBV infection occurs during early childhood and is often asymptomatic, but delayed EBV infection may result in IM in adolescents and adults. The symptoms of IM, most notably fever, sore throat, swollen glands and fatigue, are thought to be the clinical manifestation of an exaggerated T cell response to EBV infection and the release of inflammatory cytokines[30]. It has been suggested that the size of the initial viral dose of EBV may be a contributing factor in the development of IM and that adolescents may be more likely to encounter a larger viral dose through deep kissing during penetrative sexual intercourse[4]. A relationship between the level of the T cell response and the severity of IM has also been noted[31]. The difference in severity of symptoms between those infected with EBV at a young age and those infected during adolescence and early adulthood may be the difference in magnitude of the viral dose, with a smaller dose acquired by salivary contact in children than that acquired through sexual contact in adolescents and young adults[4,32]. In addition, recent genetic markers in the HLA class I locus have also been implicated in the immune-response to EBV infection in both IM [33] and HD,[7] suggesting that genetic factors may also play a role. Immunopathological mechanisms involved in IM, contrasted with those in asymptomatic primary EBV infection, have been reported[32,34]. As our findings may only be representative of cases severe enough to require hospital admission, further studies in people with IM who have not been hospitalised may be beneficial.

EBV-positive Hodgkin's disease has been found to be more common in people with a previous diagnosis of IM,[29] and an almost 100% prevalence of EBV seroconversion has been found in MS patients, as compared to a 90% seroconversion rate in the general population[34]. There is growing evidence of associations between IM and both HD [7-9] and MS[10-13]. The 'hygiene hypothesis' has been put forward as a possible explanation for a causal pathway between EBV and HD and MS. It proposes that a lack of early life infections or exposure to viral pathogens in childhood may prevent the normal processes of immune maturation, leading to increases in rates of both allergic and immune-mediated conditions, such as MS[35]. Perinatal and early life factors that may affect late exposure to infection may play a role in the relationship between these conditions.

### Principal findings

The lack of association between increasing maternal age and hospitalised IM found in the current study is important, given the trend in Western countries towards postponement of childbearing. It is now common for women to give birth well into their late 30s or early 40s, and it is reassuring that older motherhood does not seem to carry an increased risk for IM. However, although maternal age has increased over recent years, in the years covered by the study (1970-1989), most mothers were under 35 (94% in our data).

There was no association between season of birth and hospitalised IM.

Our data suggested that pre-eclampsia, and forceps delivery, had a borderline significant association with subsequent IM. There was no residual association after controlling for other factors, suggesting that confounding was responsible for these apparent associations.

Children born as one of a pair of twins had a borderline significant lower risk of developing IM (p = 0.06) than that of singletons. If this is not a chance finding, it would support studies proposing that increased sibship sizes can protect against IM (and its long term sequelae, including MS), by exposing children to viral infections early in life[36]. The reasoning, part of the hygiene hypothesis, is that children born as one of twins are more likely to be exposed to EBV infection early in life, through physical and salivary contact with their sibling, thus reducing their risk of delayed EBV infection, and therefore IM, later in life.

There is no information in the literature about marital status and IM, or delayed childhood EBV infection. Our results show that children born to single mothers had a significantly lower risk of hospitalised IM than those born to married mothers. We have no explanation for this, although one possibility is that (for a given level of severity of illness) single mothers may have had greater difficulty than married mothers in accessing hospital care. Though possible, we think that this is unlikely in that, with free access to National Health Service care, children deemed to be in need of hospital care are likely to have received it. It is possible that, though the finding was highly statistically significant, it may nonetheless have arisen from the play of chance. It is worth noting that, in the era of the pregnancies covered by this study, single motherhood was much less common in England than it is now. Previous studies have found clustering of infectious diseases within households in which an older child is present[36]. Although parity is an incomplete measure of contact with older children within the household, it was the only measure available to us. It did not come close to significance in this study. It is unlikely that the association with single mothers is confounded by parity: it persisted after adjustment for parity and, in any case, there was no association between parity and risk of IM (Tables 2, 3).

We found a modest association between IM and lower social class. It is generally held that, if anything, IM is a little more common in higher social classes[36]. However, the patients in our study are those admitted to hospital and it is possible, even likely, that typical clinical thresholds for admission of patients with IM can be influenced by patients' socio-economic circumstances. Thus, for a given level of clinical severity, it is possible that children in less favoured socio-economic circumstances may be more likely than others to be admitted to hospital. The literature is conflicting over the relationship between social class and possible sequelae of late infection with EBV and HD. Several studies have reported minimal or no effect of social class on MS [37,38] or HD[39,40]. It has also been reported that EBV-infection-associated HD is in fact more common in lower social classes,[41] although this association only reached statistical significance in females[41].

## Conclusion

In summary, the association with single motherhood deserves further study, as does the possibility that reduced contact between young children may increase the risk of IM and possibly, for a few, eventually the risk of MS or HD. Other perinatal factors studied by us, including season of birth, were not associated with an increased risk of hospitalised IM. Of some importance, late age at motherhood was not a risk factor.

## Competing interests

The authors declare that they have no competing interests.

## Authors' contributions

MJG designed the study, with input from IM and OA-M. CJW undertook the analyses. All authors contributed to the interpretation and discussion of findings. IM wrote the first draft and all authors contributed to the final manuscript.

## Pre-publication history

The pre-publication history for this paper can be accessed here:

http://www.biomedcentral.com/1471-2334/11/51/prepub
